# Supplementary material for: Sulfonic Cryogels as Innovative Materials for Biotechnological Applications: Synthesis, Modification, and Biological Activity
Source: Int J Mol Sci. 2023 Feb 2;24(3):2949. doi: 10.3390/ijms24032949 (PMC9917522; doi:10.3390/ijms24032949)
Supplement: Supplementary file 1 [file ijms-24-02949-s001.zip › ijms-2084965-supplementary.pdf]

Supplementary Materials
Sulfonic cryogels as innovative materials for biotechnological applications: synthesis, modification and biological activity

Svetlana Laishevkina[1], Tatiana Kuleshova[2], Gayane Panova[2], Elena Ivan'kova[1], Olga Iakobson[1], Anatoly Dobrodumov[1], Natalia Shevchenko[1] and Alexander Yakimansky[1] *

[1] Institute of Macromolecular Compounds, Russian Academy of Sciences, Bolshoy pr., 31, Saint-Petersburg, 199004, Russia
[2] Agrophysical Research Institute, Grazhdanskiy pr. 14, Saint-Petersburg, 195220, Russia
* Correspondence: yakimansky@yahoo.com

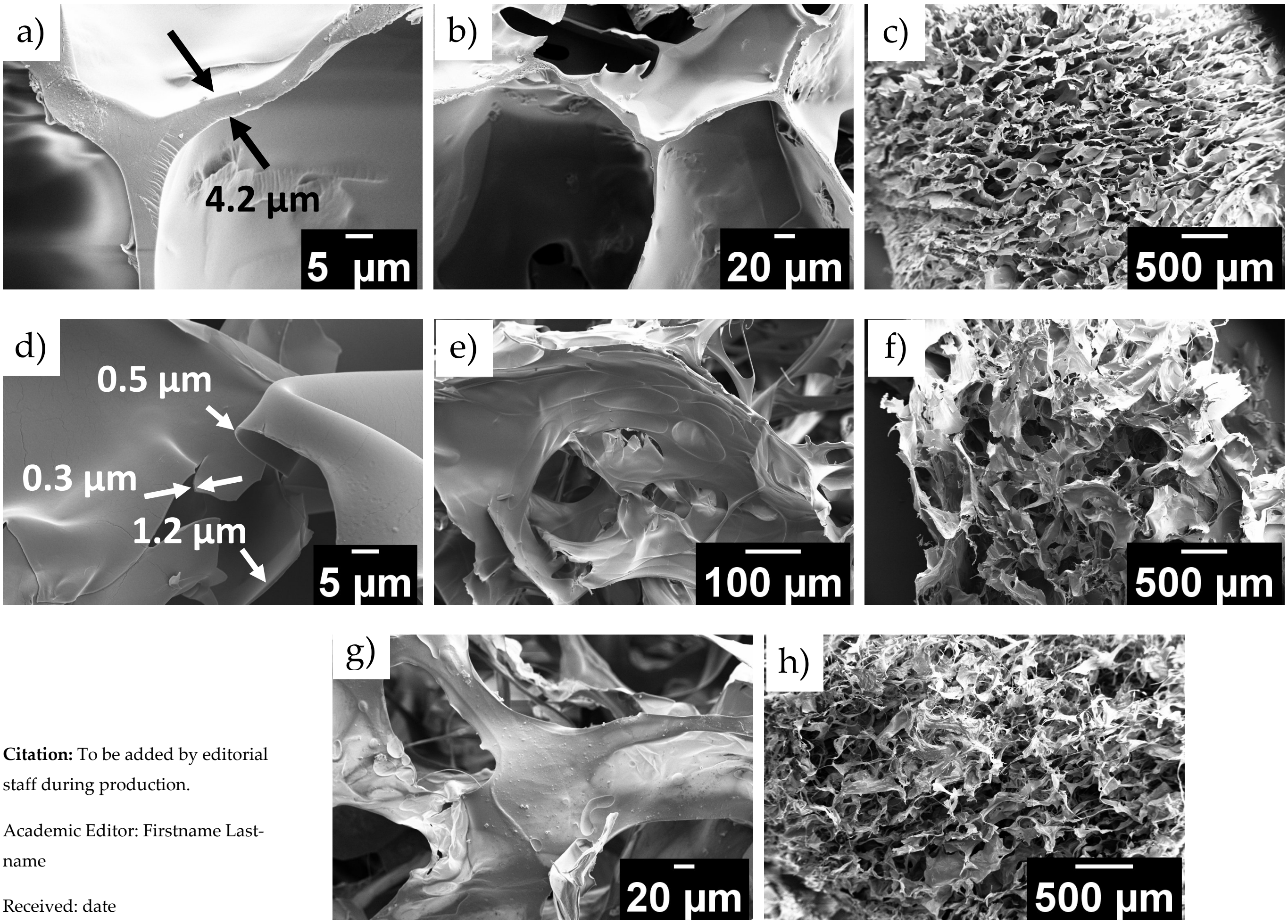

Figure S1. SEM images of the cryogel 2 (concentration of monomers is 1 mol/L) before swelling (a, b) and after swelling (c, d); cryogel 5 (concentration of monomers is 5 mol/L) after swelling (e, f); cryogel with 3 wt.% of DNDs (g, h).

Citation: To be added by editorial staff during production.

Academic Editor: Firstname Lastname

Received: date
Accepted: date
Published: date

Publisher's Note: MDPI stays neutral with regard to jurisdictional claims in published maps and institutional affiliations.

Copyright: © 2022 by the authors. Submitted for possible open access publication under the terms and conditions of the Creative Commons Attribution (CC BY) license (https://creativecommons.org/licenses/by/4.0/).

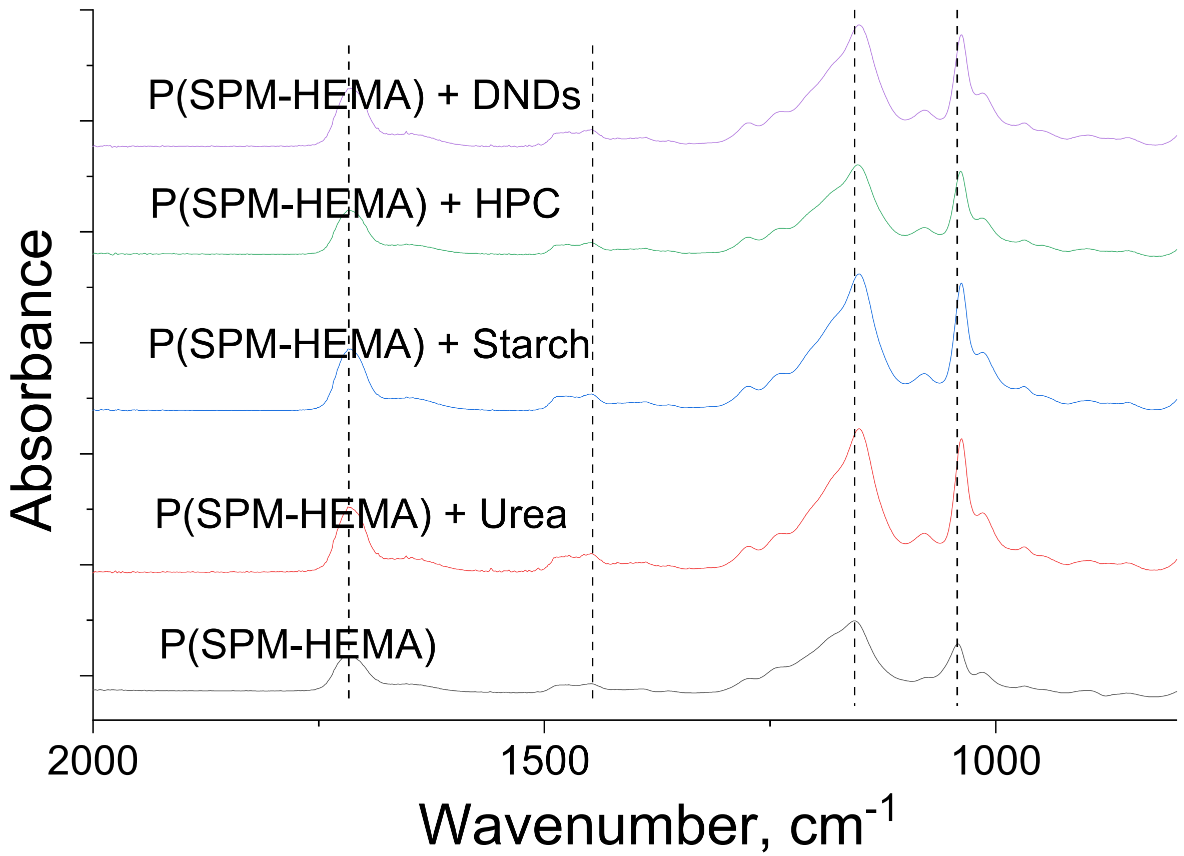

Figure S2. FTIR-spectra of P(SPM-HEMA) cryogels with additives.

(a) (b)

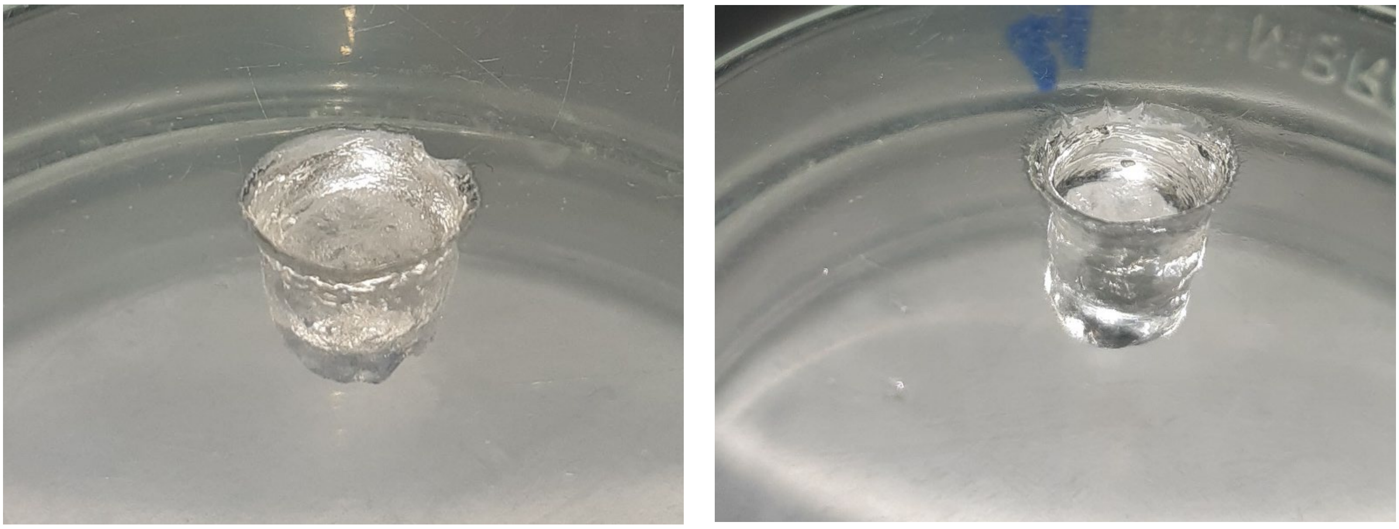

Figure S3. The photos of P(SPM-HEMA) hydrogels: initial (a) and with addition of urea (3 wt%) (b)
